# A rare case of supraventricular tachycardia induced by Infliximab: a case report

**DOI:** 10.1186/1757-1626-2-147

**Published:** 2009-10-05

**Authors:** Mukesh Singh, MM Diwan, Kiran CR Patel

**Affiliations:** 1 Department of Internal Medicine, Rosalind Franklin University of Medicine & Sciences at Chicago Medical School, Chicago, Illinois, 60064. USA; 2 Selly Oak Hospital, University Hospital Birmingham NHS Foundation Trust, Selly Oak, Birmingham, B29 6JD, UK

## Abstract

**Background:**

Infliximab, a chimeric monoclonal immunoglobulin antibody to tumor necrosis factor-α, has been established as a safe and effective treatment of rheumatoid arthritis, active and fistulising crohn's disease. Infliximab is generally well tolerated drug. The commonly reported cardiac side effects of Infliximab include exacerbation of congestive heart failure, hypotension and syncope. Symptomatic disorders of cardiac rhythm have been reported only rarely in few case reports and to the best of our knowledge, no tachyarrhythmia has been reported in past.

**Case report:**

We report the case of a supraventricular tachycardia that occurred within three hours of Infliximab infusion in a patient with rheumatoid arthritis.

**Conclusion:**

It is interesting to note that prior infusions in this patient did not precipitate similar consequences, thus, emphasising the importance of careful monitoring of patients on Infliximab therapy for possible reactions, even if prior exposures have been uneventful.

## Case presentation

A 60 year old Caucasian gentleman with rheumatoid arthritis on infliximab therapy for one year presented with acute onset palpitations. Which started within three hours after receiving the eight weekly infliximab infusion for his rheumatoid arthritis. There was no past medical history of diabtes mellitus, ischemic heart disease, hypertension or heart failure. He has never smoked cigarettes and there was no history of alcohol or illicit drug use. He had no known drug or food allergies. His medical therapy on admission included azathioprine, prednisolone, diclofenac, omeprazole, Dihydrocodeine, paracetamol, salbutamol inhaler and eight weekly Infliximab infusions.

**Figure 1:**
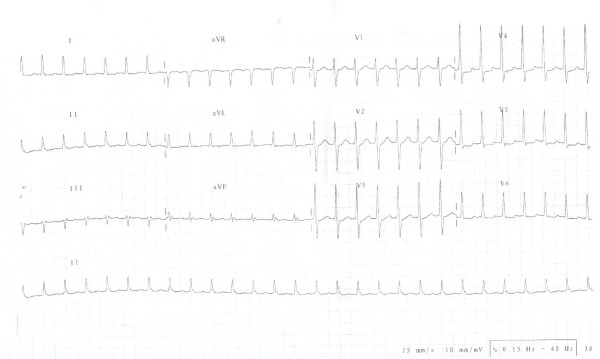
**ECG 1, The 12 lead electrocardiogram and rhythm strip showing supraventricular tachycardia on presentation**.

**Figure 2:**
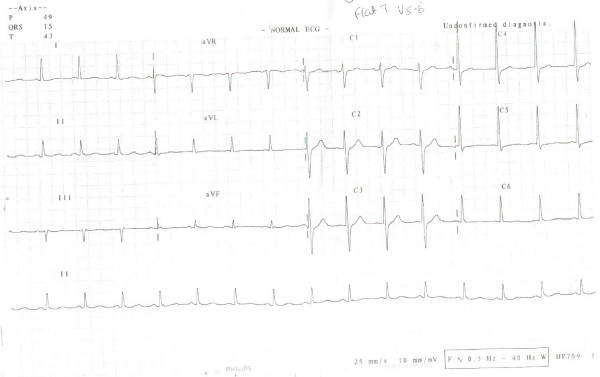
**ECG 2, The 12 lead electrocardiogram and rhythm strip after supraventricular tachycardia reverted back to normal sinus rhythm following nine milligrams of Adenosine**.

On examination he was tachycardic at 168 beats per minute. Blood pressure was 110/90 and respiratory rate of 16 per minute; oxygen saturation was 99% on room air. Systemic examination was unremarkable. The electrocardiogram (Figure 1) showed a supraventricular tachycardia (SVT), which reverted rapidly to sinus rhythm (Figure 2) with intravenous adenosine therapy (9 milligrams). His complete blood count, cardiac enzymes, troponinI, serum electrolytes, renal functions and liver enzymes all were normal. Chest X-ray did not show any evidence of cardiomegaly or pulmonary congestion and 2-D echocardiogram revealed normal left ventricular systolic and diastolic functions.

## Discussion

Infliximab is a recombinant immunoglobulin G1 kappa, human-murine chimeric monoclonal antibody that specifically and potently binds to and neutralizes the soluble TNF alpha homotrimer and its membrane-bound precursor [1]. It has been shown to be an effective treatment for moderate-to-severe active and fistulising Crohn's disease [2], and for active rheumatoid arthritis that is not responding adequately to Methotrexate therapy [3].

The safety profile of infliximab is still evolving because of its rapidly growing use and indications. Infliximab is generally well tolerated drug. An adverse event that occurs during or within 24 hours of an infliximab infusion is considered an acute infusion reaction. These acute reactions most commonly include hypotension/hypertension, dyspnea, fever, chest pain and urticarial rash [4,5].

The commonly reported cardiac side effects of infliximab include exacerbation of congestive heart failure, hypotension and syncope [5]. Symptomatic disorders of cardiac rhythm have been reported only rarely [6-9] and all these were bradyarrhythmias. To the best of our knowledge, there have been no case reports of tachyarrhythmias and this is the first case of supraventricular tachycardia caused by infliximab. Alrashid A et al recently reported a case of Crohn's disease with pulmonary involvement who had history of paroxysmal supraventricular tachycardia and this patient was treated with infliximab infusion without occurrence of cardiac arrhythmia [10].

TNF-α antagonism has been shown to adversely affect the clinical status in heart failure but the mechanism remains unknown. So, a caution is required in heart failure patients before infliximab therapy is started and close monitoring of left ventricular function during therapy is advised. In our patient, there were no clinical signs and previous history to suspect heart failure and echo showed normal left ventricle. So we can speculate it as a case of supraventricular tachycardia caused by infliximab therapy. It is interesting to note that prior infusions in this patient did not precipitate similar consequences. This underlines the need to carefully monitor patients on infliximab therapy for possible reactions, even if prior exposures have been uneventful.

The issue of whether further therapy with the drug in question should cease, should be determined by a risk-benefit evaluation and discussion of alternative therapeutic options with the patient. There is no literature available as to recurrence of such arrhythmias with further infusions. However, infliximab should be used with caution in patients with concomitant heart disease, particularly those with a history of conduction abnormalities and advanced age.

## Abbreviations

SVT: Supraventricular tachycardia; TNF: Tumor necrosis factor.

## Consent

Written informed consent was obtained from the patient for publication of this case report and accompanying images. A copy of the written consent is available for review by the Editor-in-Chief of this journal.

## Competing interests

The authors declare that they have no competing interests.

## Authors' contributions

MS and MMD drafted and wrote the manuscript. MS, MMD and KCRP participated in the care of the patient and interpretation of the investigations. All authors read and approved the final manuscript.
